# The comparison of efficacy and safety between cadonilimab (PD-1/CTLA-4) and anti-PD-1 inhibitors in patients with recurrent or metastatic cervical cancer: a retrospective real-world study

**DOI:** 10.3389/fimmu.2025.1582299

**Published:** 2025-06-02

**Authors:** Baoyue Pan, He Huang, Ting Wan, Qidan Huang, Shanyang He, Shijie Xu, Siyu Chen, Jiaxin Yin, Jundong Li, Min Zheng

**Affiliations:** ^1^ Department of Gynecology, State Key Laboratory of Oncology in South China, Guangdong Provincial Clinical Research Center for Cancer, Sun Yat-Sen University Cancer Center, Guangzhou, China; ^2^ Department of Gynecology, Guangdong Provincial People’s Hospital, Guangdong Academy of Medical Sciences, Southern Medical University, Guangzhou, Guangdong, China

**Keywords:** cadonilimab, anti-PD-1, anti-PD-1/CTLA-4, cervical cancer, immune checkpoint inhibitor

## Abstract

**Introduction:**

Cadonilimab provides substantial clinical benefits in recurrent or metastatic cervical cancer (R/M CC) in several clinical trials and meeting abstracts. However, the efficacy of cadonilimab in patients with prior failure of anti- programmed death receptor-1 (PD-1) inhibitors, as well as a direct comparison of its efficacy and safety with anti-PD-1 inhibitors, has not been reported in real-world settings.

**Methods:**

We conducted a retrospective study at our hospital, including two R/M CC patient cohorts. The first cohort consisted of 101 patients who received cadonilimab, either as monotherapy or in combination with other therapies, between July 1, 2022, and October 31, 2024. The second cohort comprised 201 patients who were treated with anti-PD-1 inhibitors (alone or in combination) but did not receive cadonilimab, between August 1, 2018, and March 31, 2024. In cadonilimab group, 4 patients received monotherapy, 13 patients received radiotherapy or surgery, 72 patients received concurrent chemotherapy, 57 patients received targeted therapy. Among anti-PD-1 group, 6 patients received monotherapy, 34 patients received radiotherapy or surgery, 127 patients received concurrent chemotherapy, 116 patients received targeted therapy. Clinicopathologic information, peripheral blood markers and treatment regimens were collected and analyzed to identify prognostic factors of cadonilimab through response rate comparison, as well as univariate, and multivariate analyses. The objective response rate (ORR) was compared between the cadonilimab and anti-PD-1 groups, stratified by PD-L1 expression. Safety data were also analyzed.

**Results:**

The cadonilimab group achieved an ORR of 59.41%, while the anti-PD-1 inhibitors group had an ORR of 44.28%. R/M CC patients with squamous cell carcinoma independently predicted prolonged progression-free survival (*p*=0.010). In patients with squamous cell carcinoma, the ORR was 60.32% in the cadonilimab group compared to 48.34% in the anti-PD-1 group. Cadonilimab was associated with a survival benefit in patients who had previously failed anti-PD-1 treatment (*p*=0.007), and showed a significantly higher ORR than anti-PD-1 inhibitors in patients with negative PD-L1 expression (69.23% vs 33.33%, *p*=0.033). The occurrence of immune related adverse events (irAEs) appeared to be associated with longer medication cycles, while severe adverse reactions were linked to shorter cycles. In addition, the cadonilimab group had a higher cumulative incidence of irAEs, including severe irAEs (12.87% *vs.* 1.99%, *p*=0.001), multi-organ irAEs, dyspnea and hypothyroidism than anti-PD-1 inhibitors group.

**Conclusion:**

Cadonilimab improved survival in R/M CC patients with previous anti-PD-1 treatment failure, achieving higher ORR in patients with negative PD-L1 expression compared to ati-PD-1 inhibitors. However, this benefit was associated with a notable increase in irAEs.

## Introduction

Cervical cancer has become the fourth most common cancer of both incidence and mortality in women since 2018 (1), with an estimated 660,000 new cases and 350,000 deaths worldwide in 2022 (2). It is now the third leading cause of cancer-related deaths in young women in the United States, a position it has held since 2019 (3).Patients with recurrent or metastatic cervical cancer (R/M CC) have a poor prognosis, with a five-year survival rate of less than 50% (4–6).

Immune checkpoint inhibitors (ICIs) have changed the guideline of cancer treatments. Anti- programmed death receptor-1(PD-1)/programmed death ligand 1 (PD-L1) and cytotoxic T lymphocyte-associated antigen-4 (CTLA-4) inhibitors are key immune checkpoint therapies, acting at distinct stages of T-cell activation. CTLA-4 inhibitors, such as ipilimumab, block CTLA-4’s interaction with CD80/CD86 on antigen-presenting cells during early T-cell activation, preventing inhibitory signaling and enhancing immune responses. PD-1/PD-L1 inhibitors, such as nivolumab and pembrolizumab, target the PD-1/PD-L1 axis in later stages, reversing T-cell exhaustion in the tumor microenvironment. By disrupting these checkpoints, ICIs restore anti-tumor immunity, offering a powerful approach in cancer immunotherapy (7).

Anti- PD-1 antibodies, have shown promising results in R/M CC patients who have failed previous lines of treatment (8, 9). Cemiplimab has demonstrated a survival benefit compared to chemotherapy in these patients, regardless of PD-L1 expression (10). The results of the KEYNOTE-826 trial recommend pembrolizumab plus chemotherapy, with or without bevacizumab, as a first-line treatment for R/M CC patients with PD-L1–positive expression (11). Based on the anti-tumor effects of anti-PD-1 therapy, additional studies have explored dual immune checkpoint inhibitors therapy, notably combining anti–PD-1 and anti- CTLA-4 antibodies. Results from CheckMate 358 indicate durable anti-tumor activity with first-line nivolumab and ipilimumab dual immunotherapy in patients with R/M CC patients, including those with PD-L1 expression on tumor cells of 1% or higher or less than 1% (12).

Cadonilimab, a bi-specific antibody targeting PD-1 and CTLA-4, demonstrated an objective response rate (ORR) of 33.0% in R/M CC patients and was approved by China’s National Medical Products Administration as a second-line treatment in June 2022 (13, 14). When combined with chemotherapy, with or without bevacizumab, cadonilimab achieved an ORR exceeding 65% (15), significantly improving both progression-free survival and overall survival (16) in R/M CC patients. Several meeting abstracts have shown that patients with R/M CC tend to benefit from combined therapies, including cadonilimab (17–19).

However, the efficacy and safety of cadonilimab in patients with R/M CC, particularly those who have failed previous treatment with anti-PD-1 inhibitors, have not been discussed in the real-world. Furthermore, there is a lack of studies comparing the differences in the efficacy and immune related adverse events (irAEs) between mono-immunotherapy and bispecific antibodies. Therefore, this study aims to analyze the clinical outcomes and adverse events in patients with R/M CC who were treated with cadonilimab.

## Methods

### Patient selection and characteristics

We initially enrolled 151 patients diagnosed with cervical cancer who received at least one cycle of cadonilimab (10 mg/kg Q3W) between July 1, 2022 and October 31, 2024, at our hospital. 33 patients lacked at least two imaging assessments, and 17 patients received cadonilimab as primary treatment without recurrence. As a result, 101 patients with R/M CC were ultimately included in this group. Demographics, histology, PD-L1 expression, treatment agents, adverse events, lab values (including the neutrophil-to-lymphocyte ratio, C-reaction protein, blood glucose and creatinine levels), and survival outcomes were collected.

We also enrolled 201 patients with R/M CC who were exclusively treated with anti-PD-1 therapy (without cadonilimab) between August 1, 2018, and March 31, 2024. We collected same data with cadonilimab group except lab values. The anti-PD-1 antibodies included pembrolizumab, nivolumab, tislelizumab, camrelizumab, sintilimab and toripalimab. The latter four antibodies were developed by Chinese companies and promoted into late-stage studies and regulatory review in China (20). The breakdown of this group was listed in **Supplementary Table 1**.

The information of these two groups was listed in **Table 1**, and the comparative baseline characteristics were listed in **Supplementary Table 2** and **Supplementary Figure 1**. We observed that cadonilimab group showed a higher proportion of patients with high ECOG scores, other type of histology (non- squamous cell carcinoma), bone metastasis than an-PD-1 inhibitors group. The comparison of baseline characteristics showed that cadonilimab had more negative factors of response.

**Table 1:** Baseline patient characteristics.

Characteristics	Cadonilimab (n=101)	Anti-PD-1 inhibitors (n=201)
Median age (year)	53 (27-73)	53 (29-72)
ECOG PS		
0	28 (27.72%)	119 (59.2%)
1	43 (42.57%)	68 (33.83%)
2	24 (23.76%)	14 (6.97%)
3	6 (5.94%)	0
FIGO stage		
I/II	39 (38.61%)	72 (35.82%)
III	37 (36.63%)	82 (40.8%)
IV	11 (10.89%)	21 (10.45%)
Unknown	14 (13.86%)	26 (12.94%)
Histology		
Squamous cell carcinoma	63 (62.37%)	151 (75.12%)
Adenocarcinoma	21 (20.79%)	26 (12.94%)
Adenosquamous cell carcinoma	3 (2.97%)	3 (1.49%)
Other	10 (9.90%)	18 (8.96%)
Unknown	4 (3.96%)	3 (1.49%)
PD-L1 expression (CPS≥1)		
Yes	43 (43.56%)	63 (31.19%)
No	13 (11.88%)	27 (13.37%)
Unknown	45 (44.55%)	111 (55.22%)
Time to recurrence (months)	15.30 (0-124.87)	–
Recurrence pattern		
Relapse involving pelvic organs	61 (60.39%)	119 (59.2%)
Relapse involving lung	36 (35.64%)	79 (39.3%)
Relapse involving liver	11 (10.89%)	22 (10.95%)
Relapse involving bone	22 (21.78%)	24 (10.94%)
Progression with anti-PD-1 treatment failure	35 (34.65%)	–
Time of immunotherapy		
First line	46 (45.54%)	90 (44.78%)
Second-line or more	55 (54.45%)	111 (55.22%)
Mono-drug	4 (3.96%)	6 (2.99%)
Combined with therapeutic regimens		
Chemotherapy	72 (71.28%)	127 (63.18%)
Target therapy	57 (56.44%)	116 (57.71%)
radiotherapy or surgery	13 (12.87%)	34 (16.92%)
≥ 10 Cycles of ICIs	37 (36.63%)	64 (31.84%)

**Figure 1:**
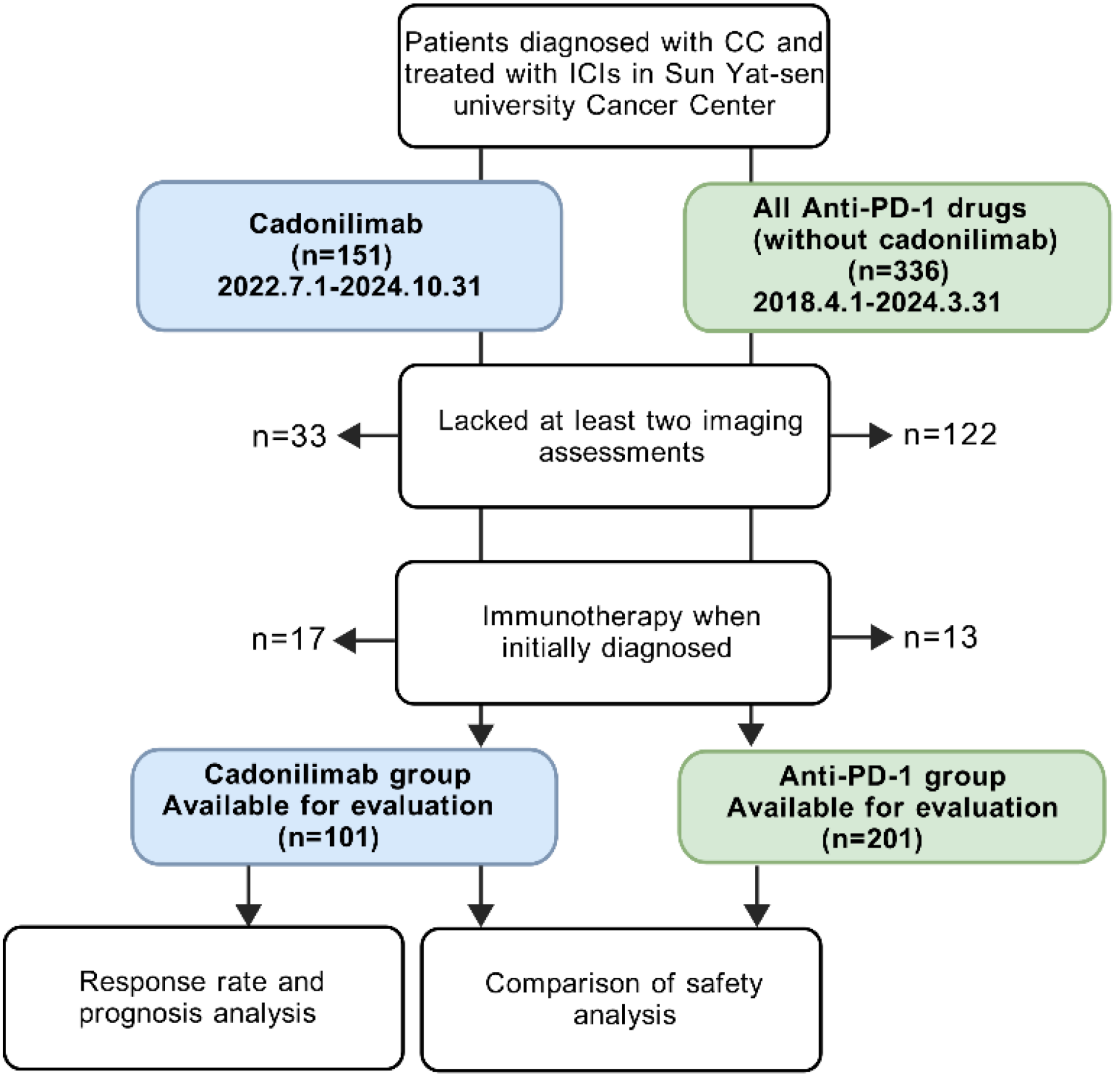
Flowchart of the patient selection process in the study.

The follow-up deadline for this study was as of December 31, 2024. This study was approved by the Institutional Review Board of Sun Yat-sen University Cancer Center (B2024-508-01) (**Figure 1**).

PD-L1 expression status mentioned in these two cohorts was analyzed with the validated PD-L1 immunohistochemistry 22C3 pharmDx assay (Agilent Technologies, Santa Clara, USA) (21).

### Failure of anti-PD-1 treatments

The failure of anti-PD-1 treatments was defined as disease progression during anti-PD-1 treatment or following an initial response to treatment. Primary anti-PD-1 treatment resistance was defined as progression during the course of anti-PD-1 treatment.

### Assessment of antitumor response and adverse events

Antitumor responses were assessed using computed tomography (CT), magnetic resonance imaging, or positron emission tomography/CT, in accordance with the Response Evaluation Criteria in Solid Tumors (RECIST) v1.1. The ORR was determined as the proportion of patients who exhibited a response to immunotherapy, including both complete response (CR) and partial response (PR). The disease control rate (DCR) was calculated as the proportion of patients who did not experience disease progression (PD). Adverse events (AEs) were recorded based on the National Cancer Institute Common Terminology Criteria for Adverse Events (CTCAE) v5.0.

### Statistical analysis

The primary endpoint was PFS. The second endpoint was ORR. Overall survival (OS) and progression-free survival (PFS), were defined as the time from the initiation of cadonilimab therapy to either the follow-up deadline or the date of death/progression, respectively. The duration from the start of the cadonilimab therapy to treatment discontinuation was named as time to discontinuation (TTD).

Pearson’s chi-square test, along with a continuity correction was conducted to analyze the relationships between the treatment response and clinical characteristics. The Kaplan–Meier method was employed to plot the survival curves, and the log-rank test was used to analyze survival rates. P < 0.05 was considered statistically significant. Statistical analyses were performed using IBM SPSS v25.0 (IBM, Armonk, NY, U.S.A.).

## Results

### Cadonilimab showed an encouraging tumor response rate

The baseline demographic and clinical characteristics of the study patients were shown in **Table 1**. The median age of these patients was 53 years. The number of patients diagnosed at an early stage was similar with those diagnosed at a late stage. More than half of the patients had squamous cell carcinoma. Only half of the patients experienced PD-L1 expression testing prior to receiving cadonilimab. Nearly one-third of the patients treated with cadonilimab had previously experienced anti-PD-1 treatment failure. Pelvic organ metastasis was the most common pattern of recurrence, observed in 60.39% of cases. Additionally, nearly half of the patients received cadonilimab as a first-line treatment following recurrence, and 60.87% patients achieved complete/partial response. ORR in cadonilimab group was 48.84% in the PD-L1 positive population and 69.23% in the PD-L1 negative population.

**Figure 2:**
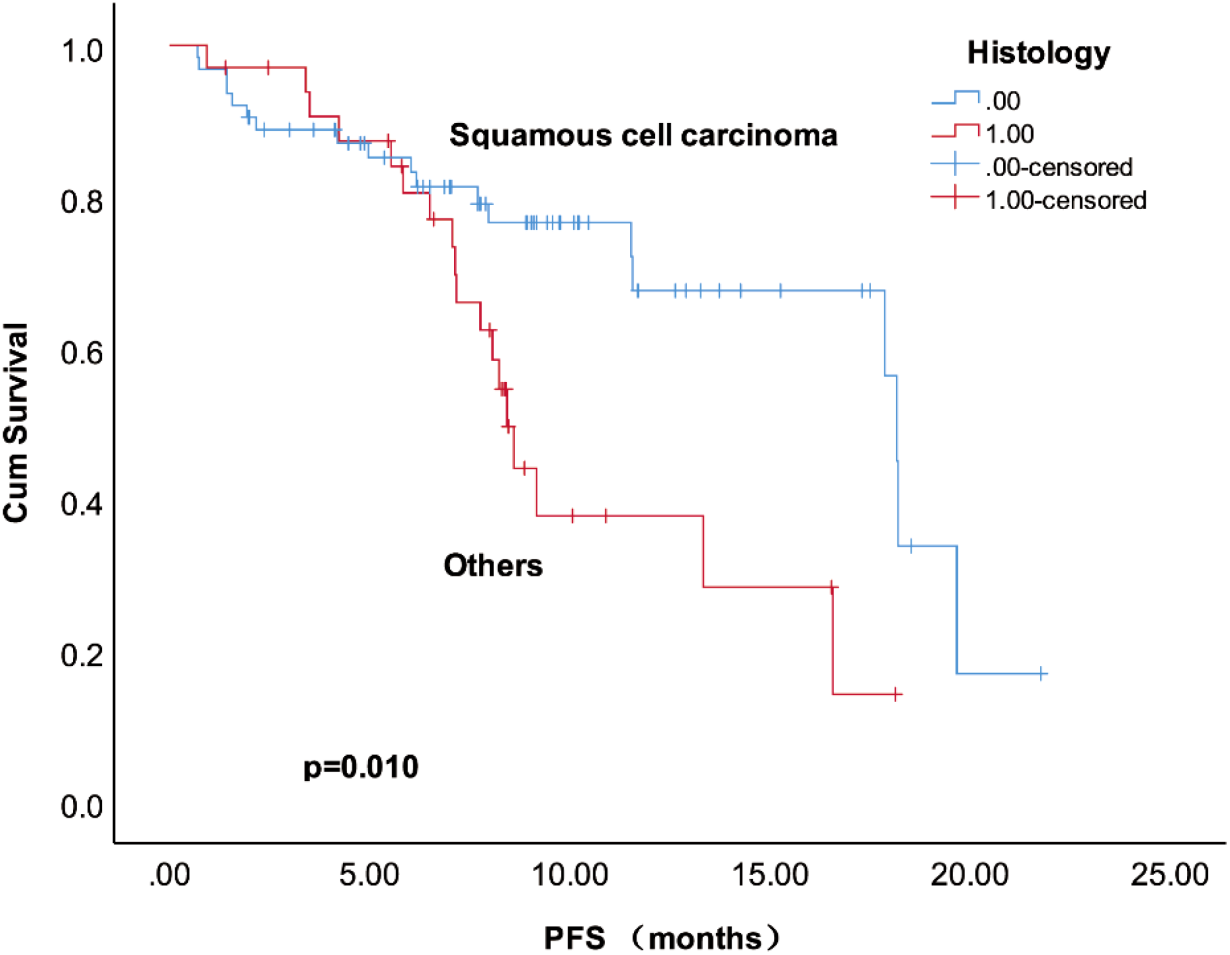
Kaplan–Meier survival curves of PFS in the R/M CC patients treated with cadonilimab.

Among the 101 patients, 60 patients (59.41%) achieved objective responses, including 4 with CR and 56 with PR. Stable disease (SD) was observed in 28 patients, while 13 patients experienced PD. The median PFS and OS were 7.97 months and 8.90 months, respectively. We analyzed the prognostic factors for PFS (**Table 2**). Squamous cell carcinoma, more cycles of cadonilimab, and low blood sugar at baseline were associated with favorable PFS. In addition, squamous cell carcinoma was the only independent factor related to PFS (**Figure 2**). Furthermore, long time to first recurrence (*p*=0.087), the combination of cadonilimab with surgery or radiation (*p*=0.052) were associated with a higher ORR although without significance (**Supplementary Table 3**).

**Table 2:** Univariate and multivariate analysis of PFS.

	Univariate analysis			Multivarite analysis		
	*p* value	HR	95% CI	*p* value	HR	95% CI
Age (≤ 50 *vs*. > 50)	0.318	1.400	0.723-2.711			
ECOG (≥2 *vs*. ≤1)	0.826	1.085	0.523-2.253			
FIGO stage						
I+II *vs*. III+IV	0.924	1.036	0.506-2.121			
Histology*						
Others *vs.* squamous cell carcinoma	**0.010**	2.451	1.239-4.849	**0.034**	2.319	1.065-5.047
Time to recurrence (> 12 vs. ≤ 12 months)	0.720	1.152	0.532-2.495			
Relapse involving pelvic organs	0.975	1.010	0.527-1.939			
Progression with anti-PD-1 treatment failure	0.138	0.587	0.291-1.187			
Beyond first-line therapy with cadonilimab	0.814	1.083	0.556-2.110			
Cycles (<10 *vs*. ≥ 10)	**0.049**	1.995	1.003-3.970	0.514	1.293	0.598-2.796
Cadonilimab combined with						
chemotherapy	0.575	1.243	0.580-2.664			
radiotherapy or surgery	0.447	0.667	0.235-1.892			
target therapy	0.651	1.163	0.604-2.238			
irAE	**0.074**	0.554	0.290-1.059	0.108	0.549	0.264-1.140
Severe irAE	0.723	0.843	0.327-2.173			
NLR (≥5 *vs*. <5)	0.480	1.302	0.626-2.705			
CRP (> 6 *vs*. ≤ 6)	0.724	1.131	0.571-2.242			
Creatinine (> 81 *vs*. ≤ 81)	0.906	1.044	0.511-2.133			
GLU (> 6.1 *vs*. ≤ 6.1)	**0.019**	2.402	1.152-5.012	0.350	1.500	0.641-3.513

*irAE, immune related adverse event.

Bold values: *p*<0.05.

### Cadonilimab showed a more favorable prognosis compared to other treatments for patients who experienced failure with anti-PD-1 therapy

We previously reported that cadonilimab showed excellent effect and manageable safety among patients with R/M CC (n=10) or endometrial carcinoma (n=4) after failure of anti-PD-1 therapy (22). Given the similar ORR observed in R/M CC patients treated with cadonilimab as a first-line (60.87%) versus second-line or beyond (58.18%), we conducted a detailed analysis of patients treated beyond the first line. Over half of these patients had experienced anti-PD-1 therapy failure.

**Figure 3:**
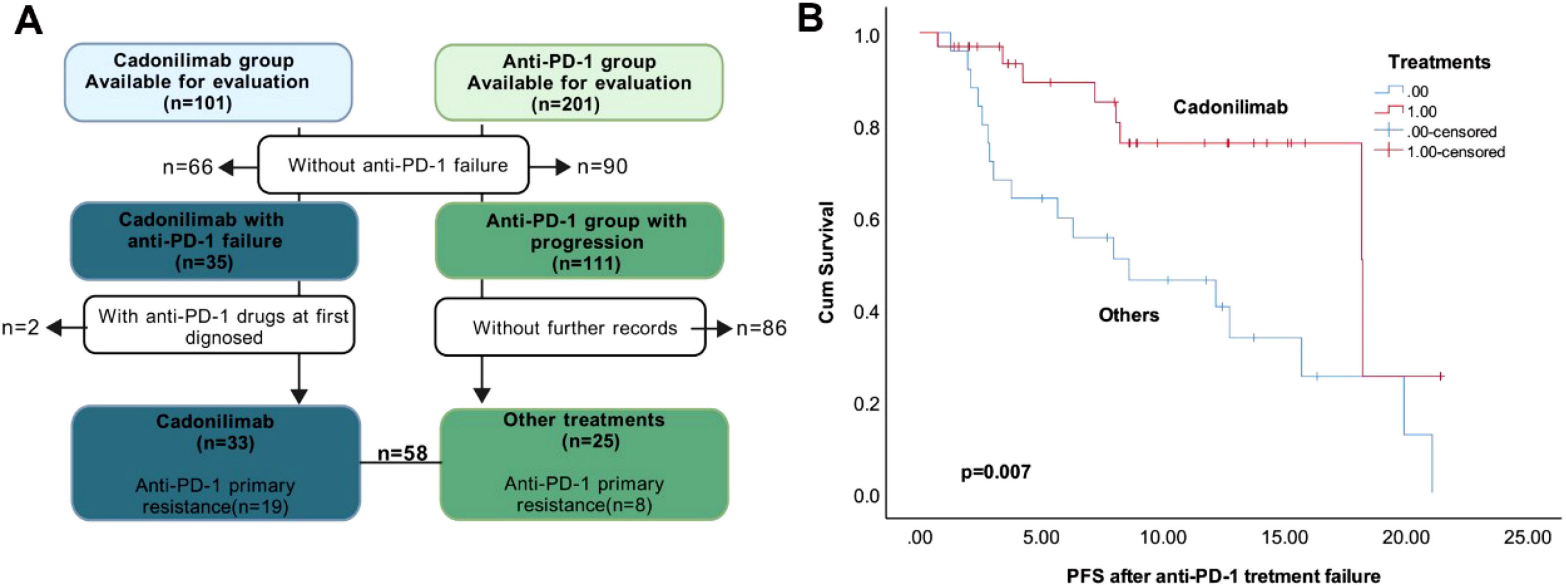
Different treatments after anti-PD-1 failure. **(A)**. The flowchart of the selection. **(B)**. Kaplan–Meier survival curves of PFS after anti-PD-1 treatment failure in the R/M CC patients.

We retrospectively analyzed 58 patients who progressed on anti-PD-1 therapy and subsequently received either cadonilimab or other treatments (anti-PD-1-based combinations or chemotherapy/radiotherapy), with detailed treatment records and imaging assessments available (**Figure 3A**). The cohort included 33 patients treated with cadonilimab, 16 who continued anti-PD-1 inhibitors combined with chemotherapy/anti-angiogenic agents, and 9 receiving chemotherapy ± radiotherapy.

Prior anti-PD-1 efficacy was comparable between the cadonilimab and non-cadonilimab groups, with ORR of 30.30% versus 36.00% (p=0.647). Although a higher proportion of patients in the cadonilimab group (57.60%) had exhibited primary resistance to prior anti-PD-1 therapy compared to those receiving other treatments (32.00%), this difference was not statistically significant (p=0.051). These findings suggest balanced baseline anti-PD-1 sensitivity between groups.

Post-progression outcomes demonstrated that cadonilimab significantly prolonged PFS compared to other treatments (p=0.007; **Figure 3B**), though no significant difference in OS was observed.

### Combination therapy including cadonilimab demonstrated higher ORR than that of ati-PD-1 inhibitors in patients with negative PD-L1 expression

**Figure 4:**
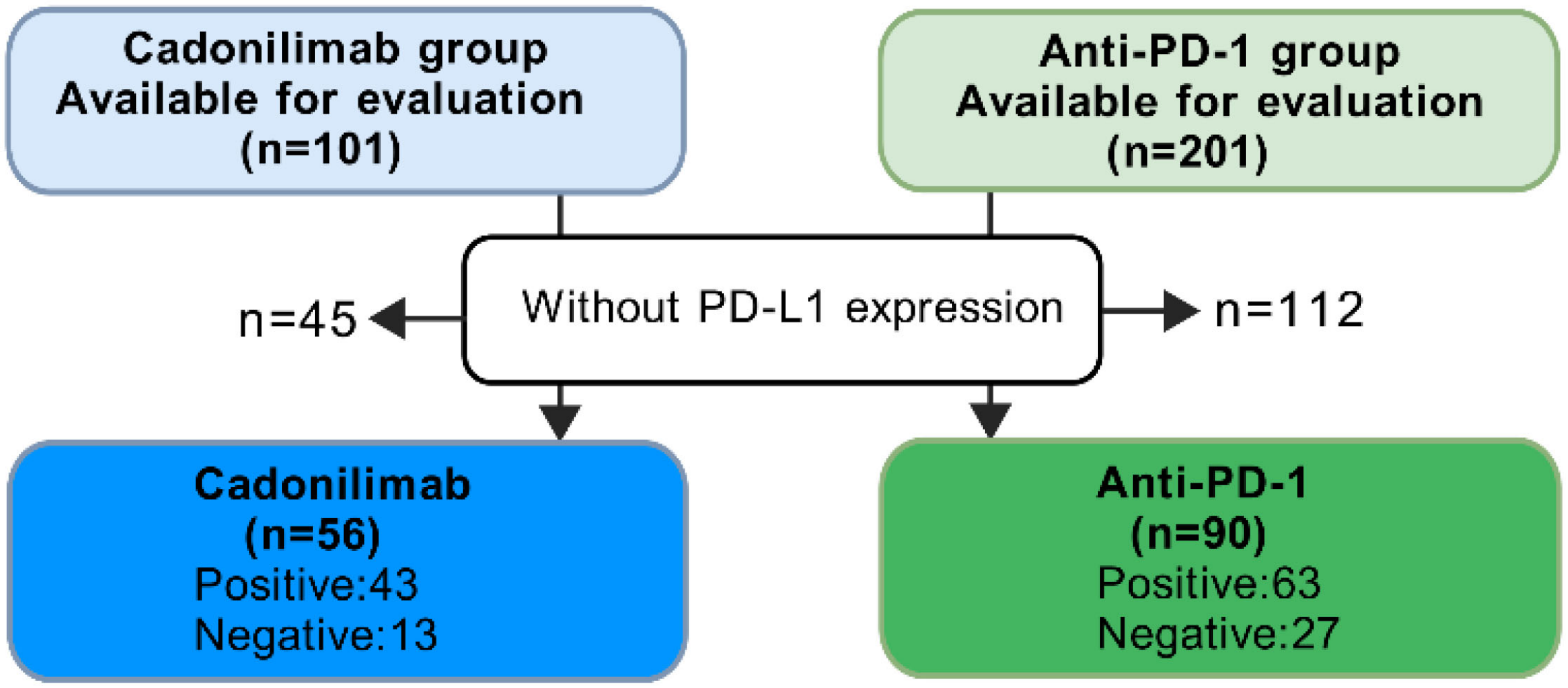
Flowchart of the patient selection process in this part.

Patients with negative PD-L1 expression had limited treatments. In the COMPASSION-03 study, cadonilimab monotherapy exhibited an ORR of 18% in cervical cancer patients with negative PD-L1 expression (13). To compare the effects of cadonilimab and anti-PD-1 inhibitors based on PD-L1 expression, we selected patients had PD-L1 expression test from these two groups (**Figure 4**). Among all PD-L1 negative patients, the ORR for anti-PD-1 inhibitors was 33.33% (9/27), while cadonilimab achieved an ORR of 69.23% (9/13). Cadonilimab showed a higher ORR in PD-L1 negative patients (*p*=0.033). In patients with positive PD-L1 expression, no significant difference in ORR was observed between cadonilimab and anti-PD-1 inhibitors (*p*=0.604, 48.84% *vs.* 53.97%).

### Safety profiles

Adverse events (AEs) were summarized for all patients treated with cadonilimab (**Supplementary Table 4**) and anti-PD-1 inhibitors (**Supplementary Table 5**). Anemia was the most common AE observed in all patients treated with cadonilimab, occurring in 30.69% of cases.

Nearly half of patients receiving cadonilimab experienced irAEs, and 13 patients discontinued cadonilimab treatment due to intolerance. We also analyzed the factors that affected TTD and found that the occurrence of irAEs, elevated creatinine levels and low blood sugar at baseline were related to longer TTD. However, severe irAEs led to shorter TTD (**Supplementary Table 6**).

In a recent study, irAEs were categorized into seven organ-based systems (endocrine, cutaneous, respiratory, gastrointestinal, hepatic, musculoskeletal, and neurological) (23). IrAEs affecting more than one organ were classified as multi-organ irAEs. A comparison of irAEs between the cadonilimab and anti-PD-1 groups (**Table 3**) revealed that while the proportions of irAEs, hyperthyroidism, rash, and elevated transaminases were not significantly different between the two groups, cadonilimab was associated with a higher cumulative incidence of irAEs over time (*p*=0.001, **Figure 3**). In addition, cadonilimab was associated with a significantly higher proportion of severe irAEs (*p*=0.001), multi-organ irAEs (*p*=0.004), dyspnea (*p*=0.001) and hypothyroidism (*p*=0.014) compared to anti-PD-1 inhibitors group.

**Table 3:** Comparison of irAEs between anti-PD-1 inhibitors and cadonilimab.

	Anti-PD-1 inhibitors (n=201)	Cadonilimab (n=101)	*p* value
irAE	84 (41.79%)	49 (48.51%)	0.267
Severe irAE	4 (1.99%)	13 (12.87%)	**0.001**
Multi-organ irAE	9 (4.48%)	14 (13.86%)	**0.004**
Hypothyroidism	18 (8.96%)	19 (18.81%)	**0.014**
Hyperthyroidism	5 (2.49%)	4 (3.96%)	0.478
Rash	13 (6.47%)	11 (10.89%)	0.180
ALT/AST elevation	21 (10.45%)	11 (10.89%)	0.906
Dyspnea	0 (0%)	8 (7.92%)	**0.001**
Elevated creatine kinase	2	2	
Pneumonitis	1	2	
Myocarditis	1	1	
Anaphylactic shock	0	1	
Colitis	0	1	

*irAE, immune related adverse event; Severe irAE: grade3–4 irAE.

**Figure 3** Cumulative incidence of irAEs according to type of treatments.

Bold values: *p*<0.05.

## Discussion

We assessed the efficacy and safety of cadonilimab in patients with R/M CC with or without prior failure of anti-PD-1 inhibitors. Patients with squamous cell carcinoma exhibited a significantly longer PFS compared to those with other histology. Cadonilimab showed survival benefit in patients who had previously failed anti-PD-1 treatment. This treatment yielded a higher ORR in patients with negative PD-L1 expression than ati-PD-1 inhibitors. However, it was associated with higher cumulative incidence of immune-related adverse events (irAEs), as well as increased rates of severe irAEs, multi-organ irAEs, dyspnea and hypothyroidism than anti-PD-1 inhibitors group.

In the COMPASSION-03 trial, cadonilimab mono-drug resulted in a 32.3% ORR in R/M CC patients who had received at least one previous systemic therapy (13). In first-line treatment for R/M CC patients, the ORR was 79.3% with or without bevacizumab in COMPASSION-13 (15). In our study, the overall ORR was 59.41%, with 60.87% in first-line therapy and 58.18% in subsequent lines. These results suggested that cadonilimab provides clinical benefits in patients with R/M CC, even in patients with progression of first line treatment. With the increasing use of anti-PD-1 inhibitors in R/M CC, many patients have developed resistance to these drugs after initially responding (24). Retreatment strategies, such as rechallenge with including anti-PD-1 inhibitors or dual-immunotherapy, are attracting significant attention (25). Previous study reported that the combination of camrelizumab, nab-paclitaxel, and apatinib showed a PR rate of 26.7% after initial immunotherapy failure (26). The combination of nivolumab and ipilimumab alleviated pembrolizumab resistance in cervical cancer (27). Cadonilimab also showed treatment potentiality for patients with anti-PD-1 resistance in a case report (28). Our findings further support the potential of cadonilimab in treating patients with anti-PD-1 resistance, with significantly better PFS than other treatments, including anti-PD-1 rechallenge, though no OS benefit was observed.

While anti-PD-1 therapies have been the standard treatment for R/M CC patients with positive PD-L1 expression (CPS ≥1), they have shown limited benefit for patients with negative PD-L1 expression, as seen in the KEYNOTE-826 trial (HR: 0.87, 95% CI: 0.50-1.52) (11, 29). To identify potential treatments for these patients, more studies focused on dual immunotherapy for R/M CC. In the CheckMate 358 trial demonstrated that nivolumab combined with ipilimumab (31% to 40% in different groups) achieved a higher ORR than nivolumab alone (26%) in first-line setting (12). This combination also showed 26% ORR in the second-line or later-line setting (30). In our study, the ORR was 69.23% (9/13) in the PD-L1 negative group and 48.84% (21/43) in the PD-L1 positive group. Further analysis suggested that cadonilimab is more effective than anti-PD-1 inhibitors in PD-L1 negative patients. However, no significant difference was observed in the efficacy between the two treatments in PD-L1 positive patients. Large sample sizes are needed to further investigate the advantages of cadonilimab in patients with negative PD-L1 expression.

The high incidence of toxicity limited the application of ipilimumab plus nivolumab in cervical cancer, as these drugs target distinct lymphocyte subtypes and act at different sites. This results in the increased incidence and broader spectrum of AEs that are difficult to manage (31). Immunotherapy-related toxicities span various organ systems and require early detection and multidisciplinary management (32). Cadonilimab, with no Fc receptor binding, showed significantly lower toxicities in the clinical practice (33). In our study, cadonilimab showed a higher proportion of irAEs and wider range of affected organs than anti-PD-1 inhibitors. In addition, severe irAEs directly led to discontinuation, although occurrence of irAEs related to a higher treatment duration.

This study has several inevitable limitations, including its retrospective design, potential selection bias, and a relatively small sample size in the cadonilimab group. Additionally, a variety of treatments were used in this heterogeneous population, and the combined chemotherapies or antiangiogenetic drugs were not standardized. Due to the economic factors, gene testing and PD-L1 expression data were not fully collected.

## Conclusion

In summary, our findings indicate that squamous cell carcinoma R/M CC patients treated with cadonilimab have favorable PFS. Cadonilimab showed clinical benefit in patients who had failed prior anti-PD-1 treatment and resulted in higher ORR in patients with negative PD-L1 expression compared to ati-PD-1 inhibitors, though with a significant increase in irAEs.

## Data Availability

The original contributions presented in the study are included in the article/**Supplementary Material**. Further inquiries can be directed to the corresponding authors.
