# Supplementary material for: The comparison of efficacy and safety between cadonilimab (PD-1/CTLA-4) and anti-PD-1 inhibitors in patients with recurrent or metastatic cervical cancer: a retrospective real-world study
Source: Front Immunol. 2025 Jun 2;16:1582299. doi: 10.3389/fimmu.2025.1582299 (PMC12171111; doi:10.3389/fimmu.2025.1582299)
Supplement: Supplementary file 1 [file DataSheet1.docx]

**Supplementary Table 1. The breakdown of anti-PD-1 therapies group**

| Anti-PD-1 drugs | Number of patients (%) | ORR | DCR |
| --- | --- | --- | --- |
| Pembrolizumab | 8 (3.98%) | 62.5% (5/8) | 87.5% (7/8) |
| Nivolumab | 2 (1.00%) | 100% (2/2) | 100% (2/2) |
| Tislelizumab | 111 (55.22%) | 38.74% (43/111) | 78.38% (87/111) |
| Camrelizumab | 33 (16.42%) | 60.61% (20/33) | 87.88% (29/33) |
| Sintilimab | 31 (15.42%) | 38.71% (12/31) | 90.32% (28/31) |
| Toripalimab | 16 (7.96%) | 43.75% (7/16) | 75.00% (12/16) |

**Supplementary Table 2. Comparative characteristics table between anti-PD-1 drugs and cadonilimab**

| Factors | *p* |
| --- | --- |
| Age (≤ 50 *vs*. > 50) | 0.297 |
| ECOG (≥2 *vs*. ≤1) | **0.000** |
| FIGO stage | 0.763 |
| I+II *vs*. III+IV |  |
| Histology |  |
| Squamous cell carcinoma *vs.* others | **0.021** |
| PD-L1 expression (CPS≥1) | 0.255 |
| Relapse involving pelvic organs | 0.907 |
| Relapse involving lung | 0.593 |
| Relapse involving liver | 0.886 |
| Relapse involving bone | **0.042** |
| Beyond first-line therapy with ICIs | 0.971 |
| Cycles (<10 *vs*. ≥ 10) | 0.211 |
| Cadonilimab combined with |  |
| chemotherapy | 0.128 |
| radiotherapy or surgery | 0.378 |
| target therapy | 0.906 |

**Supplementary Figure 1**


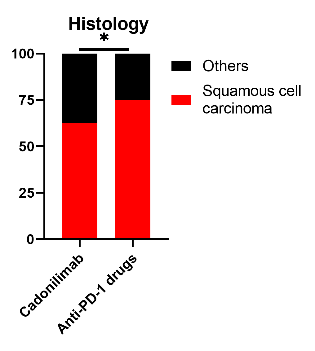

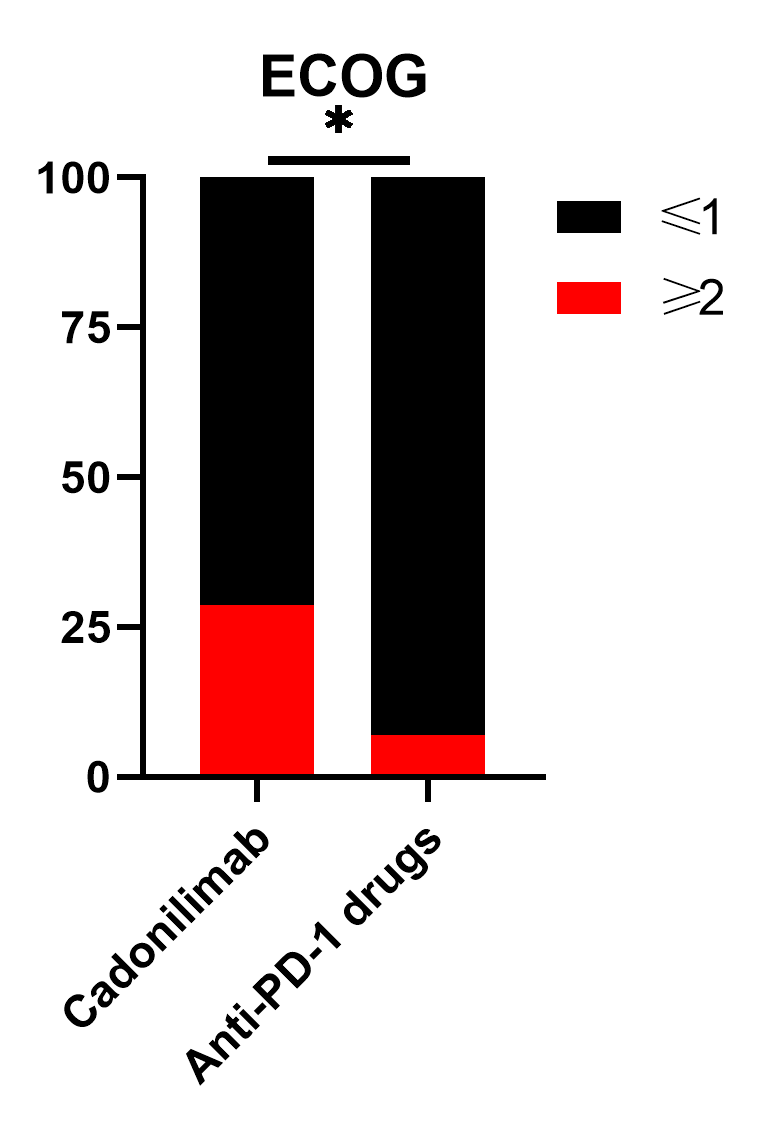

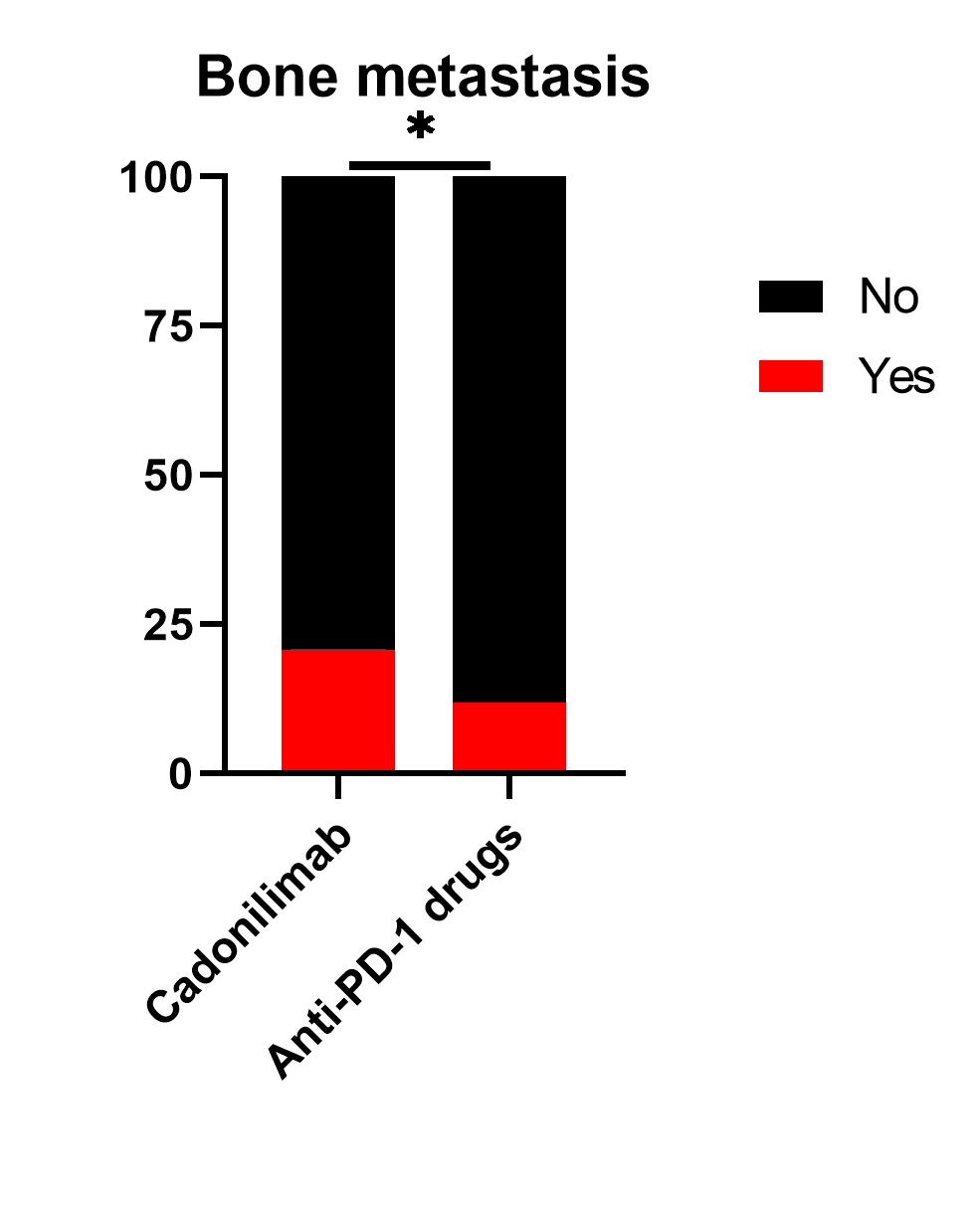


**Supplementary Table 3. Predictors of response to cadonilimab**

|  | *p* | |
| --- | --- | --- |
|  | ORR | DCR |
| Age (≤ 50 *vs*. > 50) | 0.261 | 0.154 |
| ECOG (≥2 *vs*. ≤1) | 0.427 | 0.879 |
| FIGO stage |  |  |
| I+II *vs*. III+IV | 0.249 | 1.000 |
| Histology |  |  |
| Squamous cell carcinoma *vs.* others | 0.886 | 1.000 |
| PD-L1 expression (CPS≥1) | 0.305 | 1.000 |
| Time to recurrence (> 12 vs. ≤ 12 months) | **0.087** | 0.278 |
| Relapse involving pelvic organs | 0.354 | 0.485 |
| Progression with anti-PD-1 treatment failure | 0.234 | 0.530 |
| Beyond first-line therapy with cadonilimab | 0.605 | 0.901 |
| Cycles (<10 *vs*. ≥ 10) | **0.001** | **0.041** |
| Cadonilimab combined with |  |  |
| chemotherapy | **0.084** | 0.569 |
| radiotherapy or surgery | **0.052** | 0.293 |
| target therapy | 0.187 | 0.340 |
| irAE | **0.017** | **0.049** |
| Severe irAE | 0.662 | 0.878 |
| NLR (≥5 *vs*. <5) | 0.436 | 0.342 |
| CRP (> 6 *vs*. ≤ 6) | 0.281 | 0.205 |
| Creatinine (> 81 *vs*. ≤ 81) | 0.611 | 0.552 |
| GLU (> 6.1 *vs*. ≤ 6.1) | **0.005** | **0.027** |

*irAE: immune related adverse event

**Supplementary Table 4. Treatment-related adverse events in cadonilimab group**

|  | Any Grade | Grade3-4 | Lead to treatment discountinuation |
| --- | --- | --- | --- |
|  | n | n | n |
| Any | 56 | 19 | 11 |
| Anemia | 31 | 5 | 2 |
| Hypothyroidism | 19 |  |  |
| High creatinine | 13 |  |  |
| Rash | 11 | 1 | 1 |
| ALT/AST elevation | 11 | 5 | 5 |
| Neutropenia | 9 | 3 | 2 |
| Dyspnoea | 8 | 2 | 2 |
| Hypokalemia | 6 |  |  |
| Hypoalbuminemia | 6 |  |  |
| Thrombocytopenia | 6 | 3 |  |
| Fatigue | 6 |  |  |
| Diarrhea | 4 |  |  |
| Hyperthyroidism | 4 |  |  |
| Hyperglycemia | 2 |  |  |
| Elevated creatine kinase | 2 | 1 | 1 |
| Fever | 2 |  |  |
| Fistula | 2 |  |  |
| Headache/dizziness | 2 |  |  |
| Pneumonitis | 2 | 2 | 2 |
| Palpitation | 1 |  |  |
| Anaphylactic shock | 1 | 1 | 1 |
| Myocarditis | 1 | 1 | 1 |
| Colitis | 1 |  |  |
| Myasthenia | 1 | 1 | 1 |
| Nausea or vomiting | 1 |  |  |

**Supplementary Table 5. Treatment-related adverse events in anti-PD-1 groups**

|  | Any Grade | Grade3-4 | Lead to treatment discountinuation |
| --- | --- | --- | --- |
|  | n | n | n |
| Any | 167 | 14 |  |
| Anemia | 79 | 1 |  |
| Hypothyroidism | 37 | 0 |  |
| ALT/AST elevation | **23** | 3 | 3 |
| Thrombocytopenia | 20 | 5 |  |
| Hypertension | **19** | 2 |  |
| Hand-foot syndrome | 17 | 0 |  |
| Neutropenia | 18 | 2 |  |
| Rash | 14 | 0 |  |
| High creatinine | 10 | 3 | 3 |
| Pain | 11 | 3 | 3 |
| Fatigue | 10 | 0 |  |
| Anorexia | 17 | 0 |  |
| Diarrhea | 6 | 0 |  |
| Nausea or vomiting | 8 | 0 |  |
| Hyperthyroidism | 5 | 0 |  |
| Headache/dizziness | 6 | 0 |  |
| Hyperglycemia | 5 | 1 | 1 |
| Hoarseness | 4 | 0 |  |
| Dental ulcer | 3 | 0 |  |
| Hypoalbuminemia | 4 | 0 |  |
| Constipation | 3 | 0 |  |
| Pneumonitis | 2 | 0 |  |
| Myocarditis | 2 | 0 |  |
| Creatine phosphokinase elevation | 2 | 0 | 0 |
| Fistula | 2 | 1 | 1 |
| Fever | 2 | 0 |  |

**Supplementary Table 6. Univariate and multivariate analysis of TTD**

|  | Univariate | | | Multivariate | | |
| --- | --- | --- | --- | --- | --- | --- |
|  | *p* | HR | 95% CI | *p* | HR | 95% CI |
| Age (≤ 50 *vs*. > 50) | 0.251 | 1.355 | 0.806-2.279 |  |  |  |
| ECOG (≥2 *vs*. ≤1) | 0.349 | 0.756 | 0.422-1.357 |  |  |  |
| FIGO stage |  |  |  |  |  |  |
| I+II *vs*. III+IV | 0.881 | 1.043 | 0.603-1.803 |  |  |  |
| Histology* |  |  |  |  |  |  |
| Others *vs.* squamous cell carcinoma | 0.171 | 1.441 | 0.855-2.428 |  |  |  |
| Time to recurrence (> 12 vs. ≤ 12 months) | 0.702 | 0.903 | 0.535-1.524 |  |  |  |
| Relapse involving pelvic organs | 0.575 | 0.862 | 0.513-1.448 |  |  |  |
| Progression with anti-PD-1 treatment failure | 0.658 | 0.884 | 0.511-1.529 |  |  |  |
| Beyond first-line therapy with cadonilimab | 0.306 | 0.761 | 0.452-1.283 |  |  |  |
| Cadonilimab combined with |  |  |  |  |  |  |
| chemotherapy | **0.052** | 1.909 | 0.993-3.670 | 0.141 | 1.695 | 0.840-3.422 |
| radiotherapy or surgery | 0.302 | 0.674 | 0.319-1.426 |  |  |  |
| target therapy | 0.497 | 0.840 | 0.509-1.388 |  |  |  |
| irAE | **0.058** | 0.601 | 0.355-1.018 | **0.010** | 0.425 | 0.222-0.812 |
| Severe irAE | **0.015** | 2.209 | 1.168-4.178 | **0.027** | 2.446 | 1.108-5.488 |
| Disease progression | **0.066** | 1.599 | 0.969-2.636 | 0.175 | 1.450 | 0.848-2.479 |
| NLR (≥5 *vs*. <5) | 0.317 | 1.325 | 0.764-2.300 |  |  |  |
| CRP (> 6 *vs*. ≤ 6) | **0.023** | 0.531 | 0.308-0.916 | 0.132 | 0.630 | 0.345-1.149 |
| Creatinine (> 81 *vs*. ≤ 81) | **0.004** | 0.397 | 0.212-0.745 | **0.039** | 0.488 | 0.246-0.966 |
| GLU (> 6.1 *vs*. ≤ 6.1) | **0.013** | 2.116 | 1.172-3.819 | **0.032** | 2.028 | 1.061-3.877 |

*irAE: immune related adverse event
